# Slc39a7/zip7 Plays a Critical Role in Development and Zinc Homeostasis in Zebrafish

**DOI:** 10.1371/journal.pone.0042939

**Published:** 2012-08-13

**Authors:** Guang Yan, Yuchao Zhang, Junlei Yu, Yu Yu, Fan Zhang, Zhuzhen Zhang, Aimin Wu, Xianghua Yan, Yi Zhou, Fudi Wang

**Affiliations:** 1 Group of Bio-Metal Metabolism, Key Laboratory of Nutrition and Metabolism, Institute for Nutritional Sciences, Shanghai Institutes for Biological Sciences, Chinese Academy of Sciences, Graduate School of the Chinese Academy of Sciences, Shanghai, People's Republic of China; 2 College of Animal Sciences and Technology, Key Laboratory of Swine Genetics and Breeding, Ministry of Agriculture, Huazhong Agricultural University, Wuhan, China; 3 Schools of Life and Food Engineering, Nanchang University, Nanchang, China; 4 Stem Cell Program and Division of Hematology/Oncology Children's Hospital Boston and Dana-Farber Cancer Institute, Harvard Stem Cell Institute, Harvard Medical School, Boston, Massachusetts, United States of America; Deakin School of Medicine, Australia

## Abstract

**Background:**

Slc39a7/Zip7, also known as Ke4, is a member of solute carrier family 39 (Slc39a) and plays a critical role in regulating cell growth and death. Because the function of Zip7 *in vivo* was unclear, the present study investigated the function of zip7 in vertebrate development and zinc metabolism using zebrafish as a model organism.

**Principal Finding:**

Using real-time PCR to determine the gene expression pattern of *zip7* during zebrafish development, we found that *zip7* mRNA is expressed throughout embryonic development and into maturity. Interestingly, whole mount *in situ* hybridization revealed that while *zip7* mRNA is ubiquitously expressed until 12 hours post-fertilization (hpf); at 24 hpf and beyond, *zip7* mRNA was specifically detected only in eyes. Morpholino-antisense (MO) gene knockdown assay revealed that downregulation of *zip7* expression resulted in several morphological defects in zebrafish including decreased head size, smaller eyes, shorter palates, and shorter and curved spinal cords. Analysis by synchrotron radiation X-ray fluorescence (SR-XRF) showed reduced concentrations of zinc in brain, eyes, and gills of *zip7*-MO-injected embryos. Furthermore, incubation of the *zip7* knockdown embryos in a zinc-supplemented solution was able to rescue the MO-induced morphological defects.

**Significance:**

Our data suggest that zip7 is required for eye, brain, and skeleton formation during early embryonic development in zebrafish. Moreover, zinc supplementation can partially rescue defects resulting from *zip7* gene knockdown. Taken together, our data provide critical insight into a novel function of zip7 in development and zinc homeostasis *in vivo* in zebrafish.

## Introduction

Zinc is an essential trace element required for DNA synthesis, cell division, regulation of transcription, and protein synthesis. Approximately 2000 enzymes use zinc as a catalytic cofactor [1], and zinc binding motifs are found in up to 10% of the proteins encoded by the human genome [2] including zinc-finger-containing proteins, the most abundant protein superfamily in the mammalian genome. In this regard, zinc is an essential cofactor required for the activity of numerous proteins involved in cellular signaling pathways and biological processes including growth factors, cytokines, receptors, enzymes, and transcription factors [3], [4], [5], [6]. In addition, zinc has been found to play a role in cell-mediated immunity and signal transduction, and as an antioxidant and an anti-inflammatory agent [7], [8]. It is broadly acknowledged that numerous disorders are the result of zinc deficiency such as poor appetite, growth retardation, skin lesions, mental lethargy, delayed wound healing, neurosensory disorders, and cell-mediated immune disorders [9], [10], [11].

Zinc homeostasis in single cells and in whole organism is regulated by two families of zinc transporters: zinc exporters (Slc30a/ZnT or CDF) and importers (Slc39a/Zip)[10], [11], [12], [13], [14], [15], [16], [17]. In addition, the tissue-specific expression of each zinc transporter gene, the metals, hormones and cytokines that influence their expression, and the diseases that have been linked to their aberrant expression have been elucidated [16]. There are 10 ZnT family members in mammals. Deficiency in *ZnT2* or *ZnT4* causes reduced zinc concentrations in milk in mammals [18], [19], while *ZnT5*-KO mice suffer from growth retardation and osteogenic problems [20]. ZnT5 and ZnT6 are both localized to the Golgi apparatus [21] and uniquely form a heterodimer [22]. ZnT8 is specifically expressed in pancreatic β-cells and has been identified as a novel target autoantigen in patients with type 1 diabetes [23]. Furthermore, mutation of ZnT8 is associated with glucose intolerance and type 2 diabetes [24]. The Zip family can be divided into four subfamilies, named subfamilies I and II, gufA and LIV-1, with most mammalian Zip family members being classified into the LIV-1 subfamily. The LIV-1 subfamily contains nine members (Zip4, Zip5, Zip6, Zip7, Zip8, Zip10, Zip12, Zip13, and Zip14) [17]. With the exception of Zip7 and Zip13, a feature of the LIV-1 subfamily are a potential metalloprotease motif (HEXPHEXGD) in the fifth transmembrane domain (TMD V) [17], [25]. Mutations in the human *ZIP4* gene cause the inherited disorder acrodermatitis enteropathica [26], and our previous studies identified Zip4 as a critical regulator of zinc homeostasis via a process distinct from zinc-stimulated endocytosis [27], [28]. While *Zip5* expression is restricted to many tissues important for zinc homeostasis, including the intestine, pancreas, liver and kidneys, abundance of *Zip5* mRNA is not altered in response to changes in zinc concentration [29]. Rather, Zip4 and Zip5 are both dynamically regulated by several post-transcriptional, translational, and post-translational mechanisms [30]. Interestingly, zebrafish zip6/liv1 controls the epithelial-mesenchymal transition (EMT) via activation of signal transducer and activator of transcription 3 (STAT3), suggesting that zip6/liv1 may have an important role in cell migration [31], [32]. The Zip13 molecule is involved in the bone morphogenetic protein (BMP)/transforming growth factor beta (TGF-β) signaling pathway by controlling the nuclear localization of Smad proteins [33]. Understanding of the diverse functions of the Liv1 family continues to expand as more studies are conducted in model organisms.

Zip7 (Slc39a7, Ke4) also belongs to the LIV-1 subfamily of zinc transporters [34], and has been shown to play a critical role in maintaining the intracellular balance of zinc by affecting the redistribution of zinc from intracellular stores to the cytosol [35]. While ectopic expression of Zip7 in cells results in an increase in intracellular zinc concentration [25], Zip7 is localized to the membranes of endoplasmic reticulum (ER) and Golgi apparatus, but not to the plasma membrane; suggesting that Zip7 functions to transport zinc from the ER and Golgi to the cytosol of mammalian cells [34] [36]. In addition, recent data suggests that Zip7 acts at a critical link in zinc-mediated tyrosine kinase signaling, and may be involved in breast cancer progression [25]. A recent study identified protein kinase casein kinase II (CK2) as the kinase responsible for Zip7 activation. CK2 could trigger cytosolic zinc signaling pathways through phosphorylation of Zip7 and in turn, affect proliferative responses and cell migration[37].

Although Zip7 has attracted much interest in numerous fields of research and many studies have been performed in primary cells and in cell lines, the *in vivo* functions of Zip7 have not been determined due to a lack of a *Zip7* gene knockout animal model. Zip7 orthologs include CATSUP in Drosophila [38] and IAR1 in Arabidopsis [39]. Although knockout of CATSUP in *Drosophila* is lethal, testing partial loss of function mutants revealed CATSUP could downregulate tyrosine hydroxylase, a rate-limiting enzyme for production of dopamine in the brain [38]. Furthermore, IAR1 has been suggested to transport zinc or other metals out of the ER and into the cytosol [39]. In this study, we have elucidated the *in vivo* role of zip7 in the zebrafish vertebrate model. Previous studies revealed that zebrafish eyes highly express zinc transporters *znt4* and *zip1*, and have constitutively high levels of *zip7* [40]. We extended these findings and found that zebrafish *zip7* has essential roles in eye development. Morpholino-induced loss of *zip7* (*zip7-*MO) resulted in decreased eye and head size as well as campylorrhachia. Together, our results suggest that zip7 may play an important role in eye, brain, and skeletal development by regulating zinc transport during zebrafish development.

## Results

### The expression pattern of *zip7* mRNA during zebrafish embryogenesis

**Figure 1:**
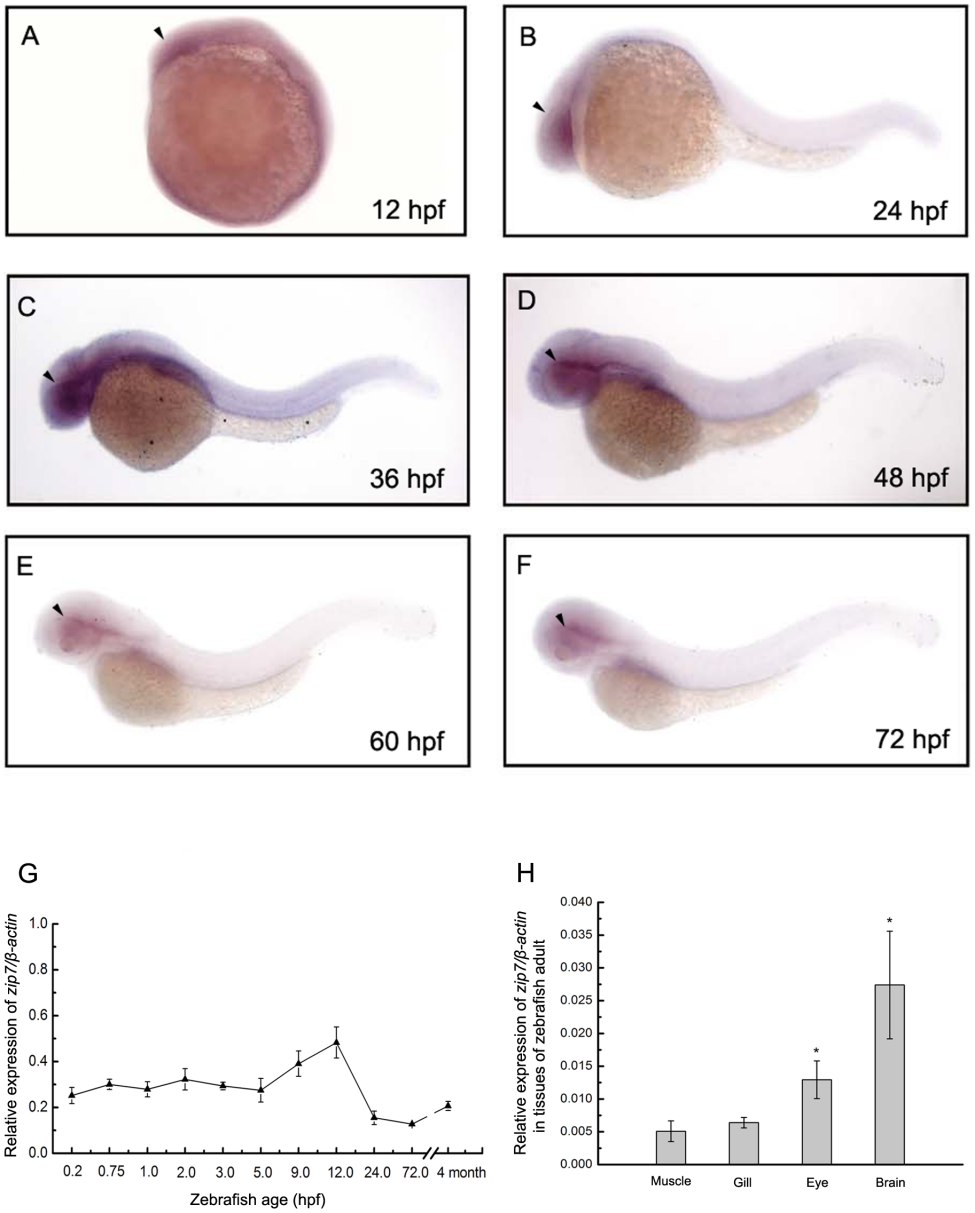
*Zip7* expression during zebrafish early embryonic development in wild type embryos and in tissues of adult zebrafish. (A–F) Lateral views (anterior to the left) of WISH of *zip7* expression at 12 hpf, 24 hpf, 36 hpf, 48 hpf, 60 hpf, 72 hpf, embryos. Arrows indicate the eye. (G) The *zip7* mRNA expression pattern assayed by qRT-PCR normalized to *β-actin* transcripts in zebrafish embryos across development and at the adult stage. (H) Normalized *zip7* mRNA expression levels in tissues of zebrafish adult. * *P*<0.05 versus muscle (1-way ANOVA, Dunnett's multiple comparison test).

We used whole-mount *in situ* hybridization to determine the spatial distribution of *zip7* gene expression during zebrafish embryogenesis and found that at early stages of somitogenesis (approximately 12hpf), *zip7* mRNA was ubiquitously expressed (Figure 1A). At 24 hpf, *zip7* mRNA transcripts were still detected in the forebrain-proximal part of retina (Figure 1B), and at later stages of zebrafish development, embryos continued to express *zip7* mRNA around the retina (Figure 1D, 1E, and 1F). Temporal changes in *zip7* mRNA expression levels during development were quantified by real-time PCR between 0.2 hpf and 120 hpf, and in adult zebrafish (4 months). Analysis of the mRNA expression pattern of *zip7* (normalized to *β-actin* mRNA) showed that *zip7* mRNA is expressed during embryogenesis and continues to be expressed in adult zebrafish (Figure 1G). In addition, *zip7* mRNA is highly expressed in brain and eye of adult zebrafish (Figure 1H).

### Silencing of *zip7* causes developmental defects in zebrafish

**Figure 2:**
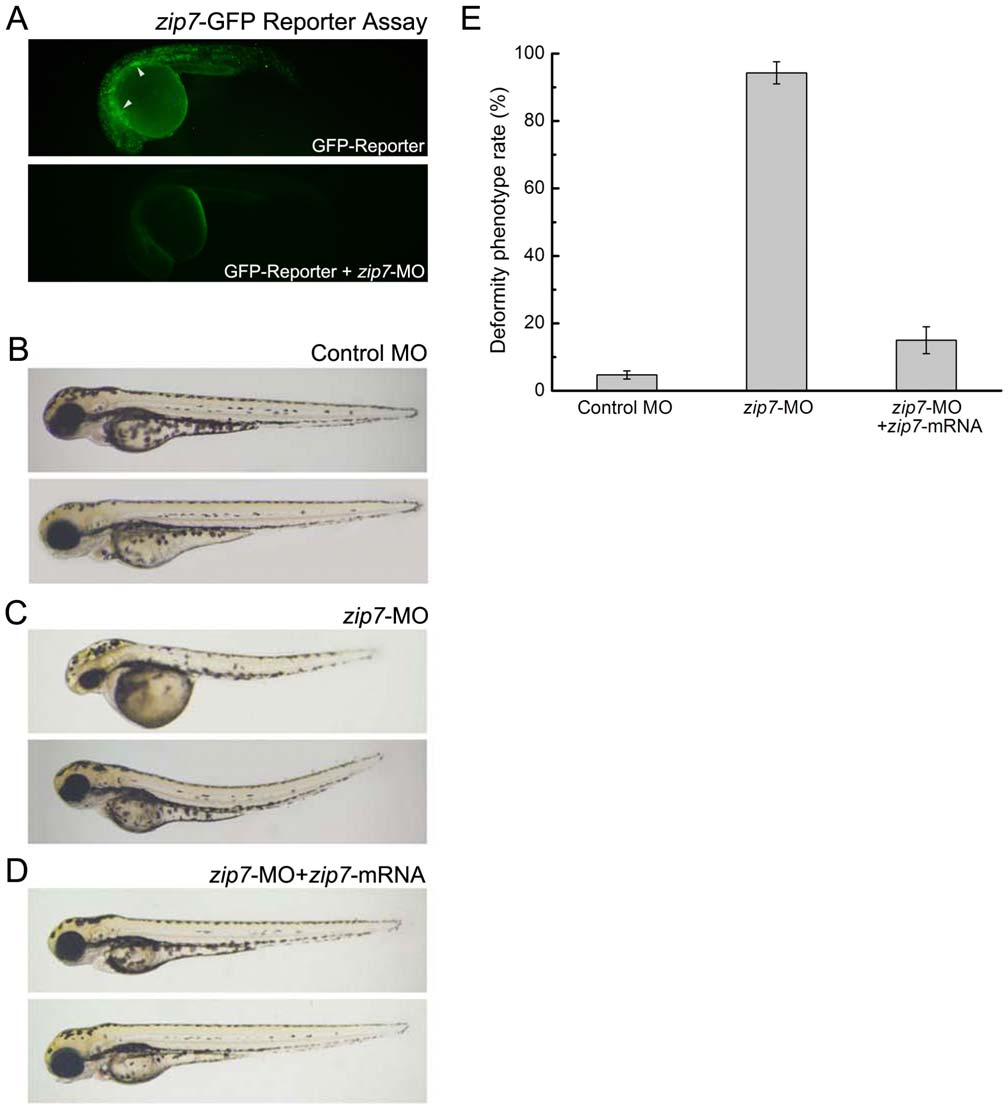
Morphological phenotypes of *zip7* loss with MO microinjection and/or *zip7* mRNA rescue at 72 hpf embryos. (A) Fluorescent images of live 24 hpf, GFP-Reporter control embryos (n = 57/60) and GFP-Reporter+*zip7*-MO morphant embryos (n = 62/62) indicate inhibition of *zip7*-GFP reporter expression (green, white arrows) by *zip7*-MO. (B–D) Lateral views (anterior to the left) of embryos at 72 hpf (B) Wild type with control-MO microinjection (2 nL, 10 ng). (C) Wild type with *zip7*-MO microinjection (2 nL, 10 ng). (D) Wild type with *zip7*-MO (1 nL, 10 ng) + *zip7* mRNA (bottom, 1 nL, 225 pg) microinjection. (E) Statistical analysis of microinjection of *zip7*-MO and *zip7* mRNA phenotypes.

We took a gene knockdown approach to study zip7 function in vertebrate development. *zip7*-MO were designed against the zebrafish *zip7* mRNA initiating ATG to block zip7 protein translation. A *zip7*-GFP reporter assay confirmed that administration of *zip7*-MO effectively blocked zip7 protein translation (Figure 2A). We tested a series of doses of *zip7*-MO and control MOs (4 ng, 6 ng, 8 ng, 10 ng, and 12 ng per embryo). While we did not detect abnormal development in embryos injected with control MO at any stage (Figure 2B), we did observe obvious developmental defects at multiple stages (24 hpf, 48 hpf, and 72 hpf) in embryos injected with *zip7*-MO. Among embryos injected with 10 ng or 12 ng *zip7*-MO, severe morphological abnormalities were observed at 72 hpf. Specifically, we observed death in a fraction of the *zip7*-MO-injected embryos, as well as many embryos with delayed growth that could not hatch and displayed a strikingly curved notochord and decreased eye size compared to controls (Figure 2C).

We then cloned the full-length zebrafish *zip7* gene and observed that injection of 225 pg *zip7* mRNA alone did not cause phenotypic changes in embryos (data not shown). To confirm the specificity of the *zip7*-MO, we co-injected 225 pg *zip7* mRNA with 10 ng *zip7*-MO and found that the developmental defects described above were partially rescued (Figure 2D). Statistical analysis (Figure 2E) showed that 72 hours after microinjection, approximately 94% of the fish displayed smaller eyes, spine bending, and head dysplasia compared to controls. Strikingly, 72 hours after *zip7* mRNA and *zip7*-MO were co-injected into fertilized eggs, the *zip7* knockdown phenotype was rescued suggesting that loss of *zip7* was specifically responsible for the developmental defects observed following *zip7*-MO injection.

### Zinc supplementation can partially rescue development defects resulting from *zip7* silencing

**Figure 3:**
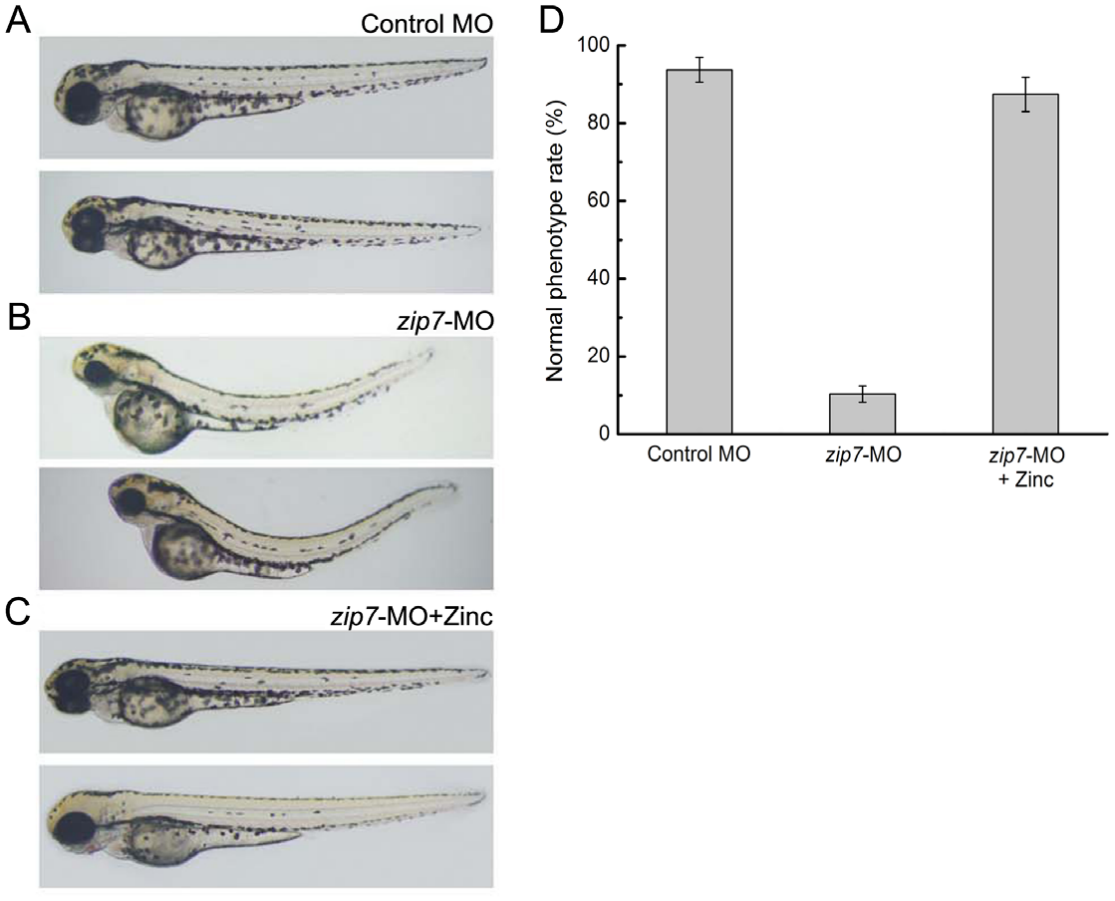
Morphologic phenotypes of *zip7*-deficient embryos and Zn^2+^ (50 µM) rescue at 72 hpf. Lateral views (anterior to the left) of embryos at 72 hpf. (A)Wild type with control-MO microinjection (2 nL, 10 ng). (B) Wild type with *zip7*-MO microinjection (2 nL, 10 ng). (C) Wild type with *zip7*-MO microinjection (2 nL, 10 ng) hatching in the presence of Zn^2+^ (50 µM). (D) Statistical analysis of microinjection *zip7*-MO and Zn^2+^ phenotype.

We next tested whether supplementation with various concentrations of zinc (Zn^2+^ 25 µM, 50 µM, 75 µM, or 100 µM) could restore normal development in hatched embryos injected with 10 ng *zip7*-MO. Strikingly, we found that, in fact, the defects we observed in zebrafish morphology during early embryonic development upon *zip7* knockdown could be rescued with zinc supplementation. In this regard, while the phenotypes of embryos at 72 hpf injected with control MO (Figure 3A) or *zip7*-MO (Figure 3B) were consistent with our previous results (Figure 2), we observed that the morphological defects in *zip7*-MO-injected embryos hatching in the presence of 50 µM zinc at 72 hpf were partially rescued (Figure 3C). Moreover, when embryos were microinjected with *zip7-*MO and then cultured in different concentrations of zinc solution, we found that in the presence of 50 µM or 75 µM zinc, approximately 80% of the *zip7*-knockdown embryos showed rescue (Figure 3D). However, the phenotype of *zip7*-knockdown embryos could not be restored in the presence of 25 µM zinc ion due to a failure to hatch, and concentrations higher than 100 µM would be toxic (data not shown). Thus, hatching in the presence of 50 to 75 µM zinc could supply an appropriate zinc environment for the development of *zip7-*MO zebrafish embryos.

### Silencing of *zip7* can result in abnormal zinc distribution in zebrafish

**Figure 4:**
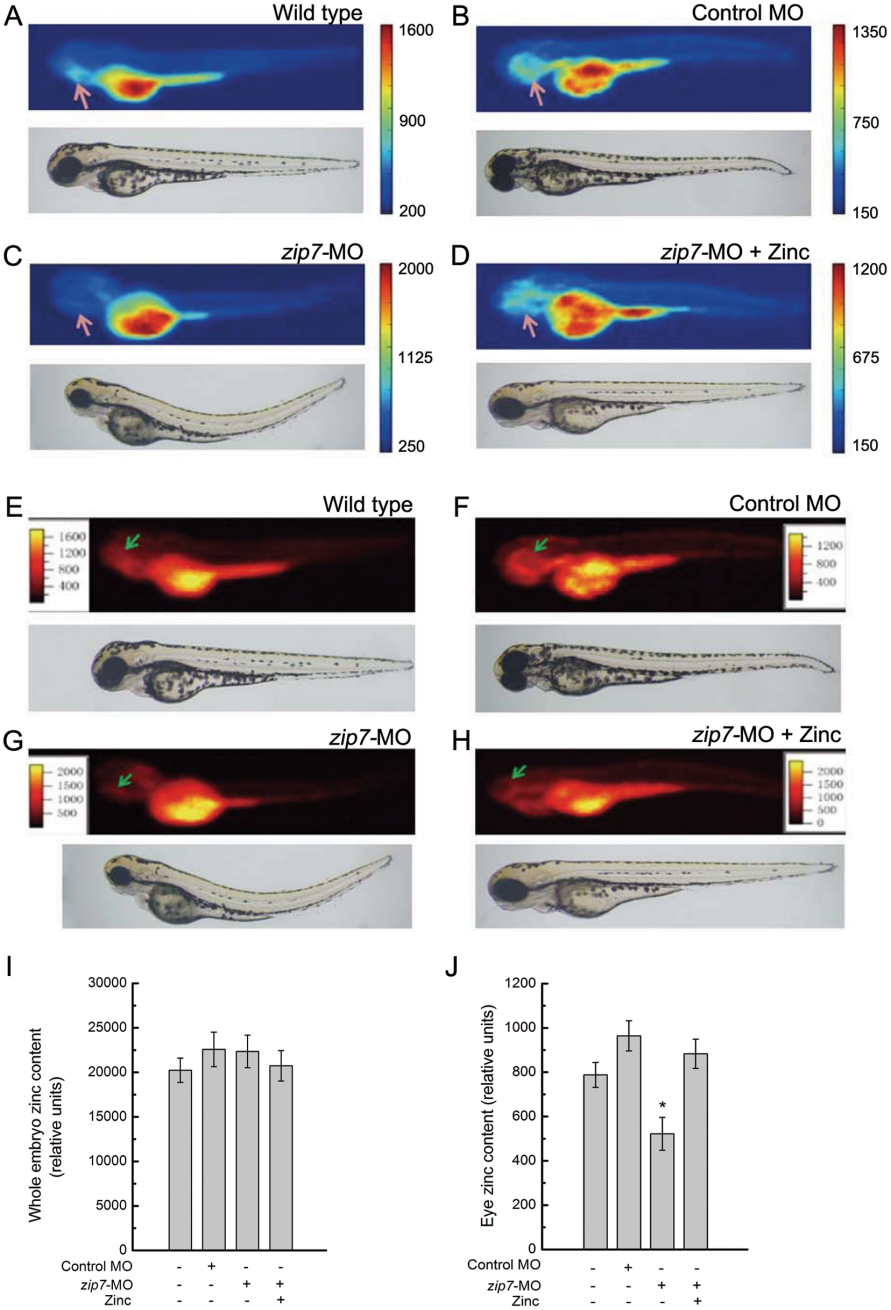
SR-XRF images and quantitative analysis of Zinc in *zip7*-Deficient Embryos and Zn^2+^ (50 µM) rescue. Lateral views (anterior to the left) of embryos at 72 hpf, arrows indicate eye. (A and E) Wild type without any microinjection, (B and F) wild type with control-MO microinjection (2 nL, 10 ng), (C and G) Wild type with *zip7*-MO microinjection (2 nL, 10 ng), (D and H) Wild type with *zip7*-MO microinjection (2 nL, 10 ng) and hatching in the presence of Zn^2+^ (50 µM). (A–D) Two-dimensional images of SSRF, (E–H) Three-dimensional images of SSRF. Quantitation and statistical analysis of zinc densities in wild type, control-MO, *zip7*-MO, and *zip7*-MO+zinc embryos (I and J). (I) Relative zinc content in the whole embryo, and (J) relative zinc content in the eye. *Statistical differences with corresponding wild type (*t* test, *P*<0.05). The zinc relative content was acquired by d4/d2/d1 (d4: photon counting of zinc correspondence, d2: electronic counting of light intensity, and d1: irradiation time).

Zinc intensity maps generated with SR-XRF showed zinc distribution and concentrations in zebrafish embryos at 72 hpf. Data were imaged at 30 µm X-Y resolution in two- and three-dimensions using Igor Pro Folder software (Figure 4). Zinc mainly distributes in eyes, gills, brain and yolk of wild type embryos. In this regard, in contrast to the images of wild type (Figure 4A and 4E) or control MO-injected embryos (Figure 4B and 4F), zebrafish with *zip7*-MO injection showed a loss of zinc in eyes (Figure 4C and 4G). However, zebrafish injected with *zip7*-MO hatching in the presence of zinc (50 µM) showed the presence of zinc in eyes (Figure 4D and 4H). Quantitation and statistical analysis showed no evident difference in the whole embryo zinc content (Figure 4I). However, it was noteworthy that zinc content in eye of *zip7*-MO was remarkably lower than wild type and control MO, while addition of exogenous zinc restored it to a normal level (Figure 4J). Together, these data indicate that zip7 could play a critical role for zinc transportation in the eyes.

### Silencing of *zip7* affects the expression of other zinc transporters

**Figure 5:**
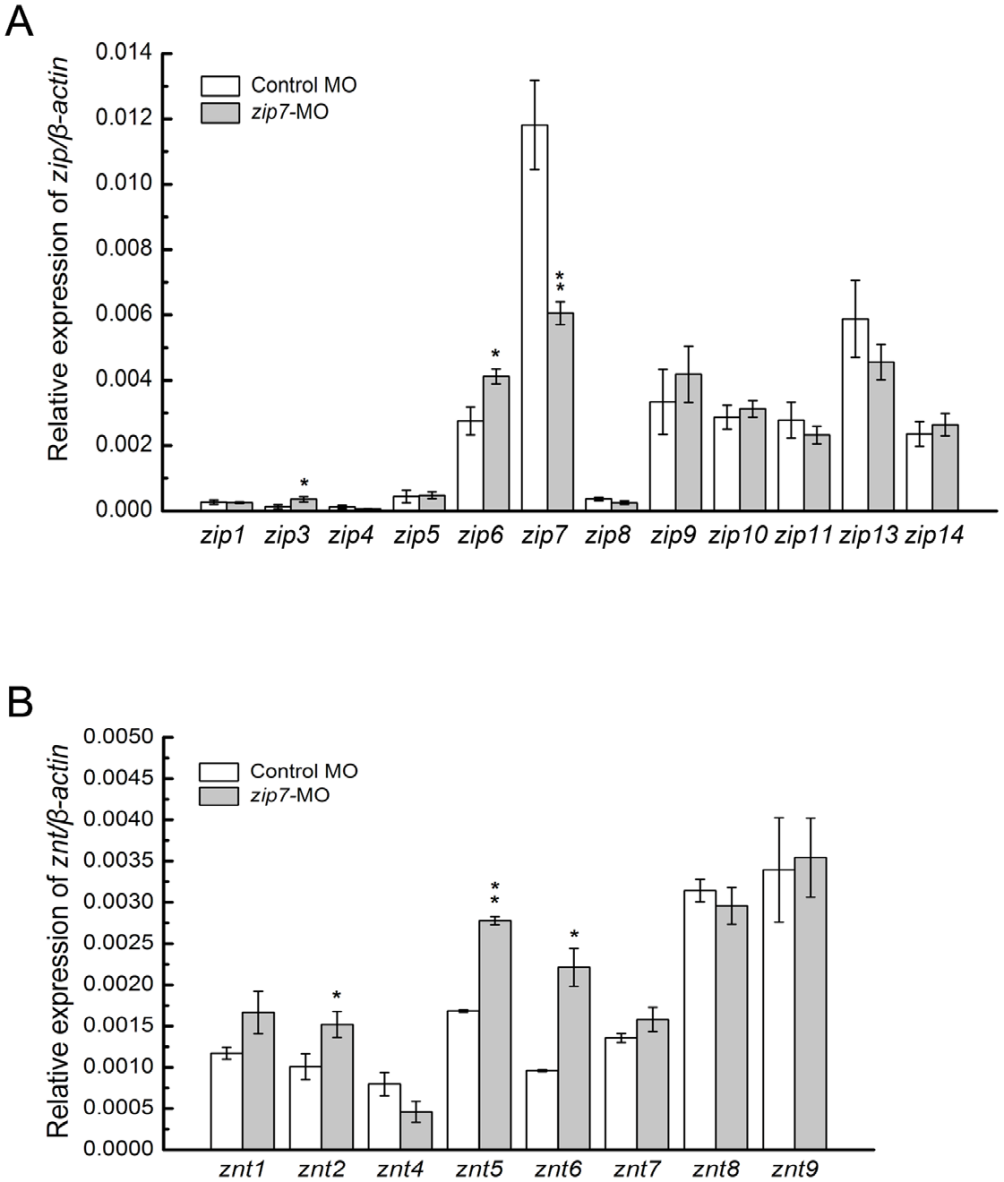
The expression of *zip* and *znt* family members in *zip7*-MO embryos. (A) Relative mRNA levels of *zip* family members in wild type embryos and *zip7*-MO embryos at 3 dpf. (B) Relative mRNA levels of *znt* family members in wild type embryos and *zip7*-MO embryos at 3 dpf. For qRT-PCR analysis, results were normalized to the internal control, *β-actin*, and presented as relative expression level calculated by the 2^ΔΔ^Ct method. Results are presented as mean ± SEM. * *P*<0.05, ** *P*<0.005.

We analyzed the expression levels of two zinc transporter family members, the *zip* and *znt* families, in *zip7*-MO and control MO embryos at 3 dpf (Figure 5A and B). Compared with the control MO embryos, the *zip7* transcript level in *zip7*-MO embryos decreased markedly. However, the expression of *zip3*, *zip6*, *znt2*, *znt5* and *znt6* increased significantly. The transcript levels of other zip and znt family members were not different between *zip7*-MO and control MO embryos.

## Discussion

In the present study, we investigated the function of Zip7 in development using zebrafish as a vertebrate model organism. In the zebrafish model system, several experimental approaches can be utilized, including RNA *in situ* hybridization, morpholino injections, and analysis of mutant and transgenic fish lines. Incorporation of these diverse approaches can lead to improved understanding of the *in vivo* regulation of key molecular pathways with conserved roles in vertebrate zinc homeostasis. For example, results from a study of zip6/liv1 using the zebrafish model suggest that zip6/liv1, as a downstream target of STAT3, is required for nuclear translocation of the Zn-finger transcription factor Snail, which regulates the epithelial-mesenchymal transition (EMT) during early zebrafish development [31].

The mouse Zip7 gene was discovered while characterizing genes in the major histocompatibility complex on chromosome 17 [41]. Human ZIP7 was mapped to the HLA class II region on chromosome 6 [42]. However, the precise function of Zip7 in whole body zinc homeostasis is not clear. Our results using RT-PCR revealed that in zebrafish, *zip7* began to be expressed early in embryonic development, and its expression continued throughout embryonic development and into maturity. These results were further confirmed by a whole-mount *in situ* hybridization assay that showed *zip7* expression gradually decreased and became restricted to developing embryos, and in adult zebrafish was found highly *zip7* expression in the brain and eye. Previous study also found *zip7* was mainly expressed in eye, brain, ovary, liver, gill and intestine in zebrafish[40]. The expression pattern of *zip7* may suggest that zip7 performs a specific function in these organs. In addition, the mouse and human *ZIP7* mRNAs were also detected in many cDNA libraries including embryo, mammary gland, ovary, uterus, cervix, testis, prostate, tongue, larynx, stomach, pancreas, bladder, eye, pituitary, bone, bone marrow, skin, and peripheral nervous system [36]. According to the human *ZIP7* gene expression atlas available in BioGPS (http://www.biogps.org/#goto=genereport&id=7922), *ZIP7* is highly expressed in prostate, pituitary gland, retina, smooth muscle, lung and colon, which is similar to zebrafish *zip7* expression.

Furthermore, we analyzed embryos lacking *zip7* using antisense morpholino (*zip7*-MO) oligonucleotides (MOs), which are the most widely used anti-sense knockdown tools in the zebrafish (*Danio rerio*) community. MOs are typically employed as oligomers of 25 morpholine bases that are targeted via complementary base pairing to the RNA of interest. A neutrally-charged phosphorodiamidate backbone results in molecules with high binding affinity for RNA, thereby facilitating steric hindrance of proper transcript processing or translation [43]. In our studies, embryos receiving injections of z*ip7*-MO displayed retarded embryonic development accompanied by smaller heads, smaller eyes, shorter palates and spinal lordosis, whereas the injection of control-MO did not result in any obvious phenotype. We also carried out rescue experiments in which we co-injected *zip7*-mRNA with *zip7*-MO, and found that the *zip7* mRNA expression was comparable to that found in wild type embryos. These results suggest that zip7 plays a critical role during zebrafish embryonic development.

Furthermore, we explored how Zip7 affected the developmental process in zebrafish through zinc rescue experiments. Addition of 50 µM zinc to hatching *zip7*-MO-injected embryos resulted in normal development, which indicated that zip7-mediates development through zinc^+^ instead of directly influencing other signal pathways. To further verify our results, we measured the distribution of zinc in whole embryos by Synchrotron radiation microbeam X-ray fluorescence (SR-XRF). SR-XRF is highly specific and sensitive for identification, characterization, and distribution analysis of metals and nonmetals in a given cell type or tissue [44]. SR-XRF is one of the few techniques capable of providing spatially resolved *in vivo* metal abundance data on a sub-micrometer scale, without the need for chemical fixation, coating, drying or even sectioning of samples [45]. The SR-XRF has been used to detect the relative contents and distributions of many trace metals in biological samples [46]. We found that the distribution of zinc between *zip7*-MO fish and wild type fish was highly different. Compared with the wild type fish, the distribution of zinc in *zip7*-MO-treated embryos was disordered and severely reduced in eyes. Quantitation and statistical analysis showed no significant difference in the whole embryo zinc content, while zinc content was severely reduced in the *zip7*-MO eye. These data indicate that *zip7* is a critical zinc transporter in zebrafish eye and is essential for zinc homeostasis during eye development. In contrast, the distribution of zinc in *zip7*-MO embryos hatching in the presence of 50 to 75 µM zinc resembled that observed in wild type embryos. This result suggested *zip7*-MO zinc content could be restored to normal levels by exogenous zinc supplementation, which was critical for zebrafish embryonic development.

Taken together, our results show that Zip7 has a very vital effect on embryonic development by regulating the absorption and distribution of zinc, however the specific details of this process remain unclear. We continued to detect the expression of other zinc transporters when *zip7* was silenced. qRT-PCR analysis revealed the expression of *zip3*, *zip6*, *znt2*, *znt5* and *znt6* were significantly increased in the absence of *zip7*. Zip3 localizes to cell bodies of the retina that also express PKC (protein kinase C) [47]. Zip6 localizes at the plasma membrane of rat neurons, suggesting a role for Zip6 in neuronal zinc uptake [48], and Zip6 has been shown to control the epithelial-mesenchymal transition in the zebrafish gastrula organizer [31]. ZnT2 expression was restricted to tissues with unique zinc requirements, such as mammary and prostate glands, where it mainly localized to the zymogen granules. In addition, ZnT2 expression level could be affected by dietary zinc content [49], [50]. ZnT5 and ZnT6 have both been shown to localize to the Golgi apparatus, and often form hetero-oligomers that function to activate alkaline phosphatases in the early secretory pathway [21], [51]. Together, these studies show that the same tissues or organelles expressing *zip7* co-express *zip3*, *zip6*, *znt2*, *znt5* or *znt6*. Silencing of *zip7* could change zinc levels and affect the expression of other zinc transporters, ultimately leading to a more serious zinc imbalance. Exogenous zinc supplementation could rectify this vicious circle, and other zinc transporters could execute their normal roles. Thus, the defect caused by *zip7* silencing can be compensated for. Previous studies revealed that zinc is essential for normal cell growth and development and is involved in protein, nucleic acid, carbohydrate and lipid metabolism, as well as in the control of gene transcription, growth, and differentiation. Intracellular zinc signals are classified into transcription-independent early zinc signaling (EZS) and transcription-dependent late zinc signaling (LZS) [52]. Many cytosolic proteins may have zinc-binding potential are expected to be closely involved in a wide range of physiological responses including development, immune function, cancer progression, and hard and connective tissue disorders [5]. Zinc itself affects a variety of signaling molecules including PKC, Ca^2+^/calmodulin-dependent protein kinase II, Erk1/2, cAMP-dependent protein kinase, protein tyrosine phosphatase, and caspase-3[5]. In addition, zinc also acts as an intracellular second messenger [3], [5]. It has been suggested that ZIP7 protein is localized to the Golgi apparatus and the endoplasmic reticulum, which are critical organelles in the redistribution of zinc from intracellular stores to the cytosol [36], [53]. As such, zinc release has downstream effects on cell signaling and hence, zinc is indeed a second messenger. An important component of zinc action in cells is the ability to inhibit protein tyrosine phosphatase activity, resulting in activation of mitogen-activated protein kinases, such as ERK1/2, c-Jun N-terminal kinase, and p38, as well as the tyrosine kinases Src and epidermal growth factor receptor. Thus, Zip7 is a central hub in cell signaling, regulating cell growth and differentiation as well as embryonic development [5], [25].

In summary, our results reveal that zip7 plays indispensable roles in maintaining zinc homeostasis and organism development especially in eyes, brain, and gills. These findings will be helpful for the understanding of mechanisms of zinc homeostasis and diseases resulting from defects in proper zinc homeostasis.

## Materials and Methods

### Fish husbandry and embryo preparation

Adult male and female zebrafish (*Danio rerio*) were maintained under a 14 hour light/10 hour dark cycle at 28.5°C with recirculating deionized water. Embryos were collected from natural matings and staged matings according to Kimmel [54]. All zebrafish experimental protocols were approved by the Institutional Animal Care and Use Committee of the Institute for Nutritional Sciences, Shanghai Institutes for Biological Sciences, and Chinese Academy of Sciences.

### qRT-PCR analysis

Pooled embryos or adult tissues were homogenized in TRIzol Reagent (Invitrogen) to extract total RNA according to the manufacturer's instructions and treated with DnaseI (Promega). RNA concentration and purity were assessed by spectrophotometry. 2.0 µg of RNA was reverse-transcribed with M-MLV reverse transcriptase (Promega) and oligo (dT) 18 primers (Takara) as recommended. PCR was performed by using CFX96™ Real-Time System (Bio-Rad) and iQ™ SYBR Green Supermix (Bio-Rad) as described in the manufacturer's manual. The reaction proceeded as follows: 95°C for 5 min, 40 cycles of 94°C for 30 s, 60°C for 30 s and 72°C for 30s. *β-actin* was used as an internal reference to normalize the PCR reaction. Primer sequences are listed in Table 1. The primers for genes (*zip1*, *zip3*, *zip4*, *zip6*, *zip9*, *zip11*, *zip13*, *znt1*, *znt2*, *znt4*, *znt5*, *znt6*, *znt7*, *znt8* and *znt9*) have been reported previously [55].

**Table 1:** Primers for qRT-PCR analysis.

Gene name	Forward Primer (5′–3′)	Reverse Primer (5′–3′)
***zip7***	TTGGTCTGTGGGTGCTAGGT	AGCAGAGGGAGAGTGGGAAT
***zip1***	GGTGAGAGTTGGAGCTCTGG	AGTGGGAAGCCATCATCAAG
***zip3***	CGTATACGGCTGATGTGGTG	AGGCCTGCTGTAAACCACTG
***zip4***	CAGACATGCTTCCTACGCTG	GCCCGATCTGGTCTTCATAA
***zip5***	TGCAACGTCTGTTCCTTCAG	TCCCAAACCCAAACTACCAA
***zip6***	GTCATCATGGGAGACGGACT	GGCAAAATCACCGAGTTCAT
***zip8***	TCCCCGCCTGCCCTTACACTT	CAGTCCAATGAAGTACATCAAAACTT
***zip9***	TCGGAATGTGACGAGCCTTCGC	ACATGTATCCTCGGAGATCGCGTG
***zip10***	TCACCTGCACATGGTGTTCT	ACATCCAAACCCATCCTGAA
***zip11***	TCAGGCCCTGCTGGGGACTC	GCCCACAGCCACTGGGAGGA
***zip13***	GGAGACCAACCCAAGGAACT	GTCTTTGGGAGGGTGACAAA
***zip14***	TAGGGGATGTTCGAGGTCAG	T CGCTCGTCTATACGGGACT
***znt1***	GAAGGCTGCCGATATGTGTC	AGGACATGCAGGAAAACACC
***znt2***	TCGGCTGGCACAGATCAGAGATT	ACCGTGGCCCACAGGACTCA
***znt4***	CATCCTGCTGGAGGGTGTA	CTGCAGTTGTACCGTGCAGT
***znt5***	TATCTCCAGTGGGAAGCTGG	ATCACTGCACACCCCATTTT
***znt6***	CCATCGCTCCGTCCTG GGGA	ACCGCCAGCACCTCGAAACG
***znt7***	CCCTTCCTGAATGCTACCAA	CACCGACCTGTGTGAAGATG
***znt8***	ATCGTCTTGATGGAAGGCA	TTTCTCGAAGCACCTCCTGT
***znt9***	CCTGTTTTGGTTGGCAAAGT	GAATGCTCTCTGCCTTCGTC
***β-actin***	CTCTTCCAGCCTTCCTTCCT	CTTCTGCATACGGTCAGCAA

### Plasmids constructions

The *zip7* coding region was amplified from whole-genome cDNA and cloned into the pCS2+ vector to generate *pCS2+-zip7*. Injection of *zip7* mRNA (*in vitro* transcribed by mMESSAGE mMACHINE® High Yield Capped RNA Transcription Kit) was performed to rescue *zip7* knockdown. For whole-mount *in situ* hybridization studies, a PCR-amplified region containing 431 bp of the last exon and the 3′UTR of *zip7* was inserted into the pCS2+ vector to generate the *pCS2+-zip7-*probe.

### Microinjection of morpholino-oligonucleotides (MOs) and mRNA

Antisense morpholino oligonucletides against *zip7* mRNA were designed and synthesized by Gene Tools, LLC. The sequences of the translational blocking *zip7*-MO were 5′-GCGATTTGCTAAAGACCCTCATTGT-3′ (−3 to −22, using the nucleotide of the start codon as the reference). The sequence of the mismatched control MO for *zip7* was 5′-CCTCTTACCTCAGTTACAATTTATA-3′. The dosage for morpholino injection was 10 ng per embryo.

Capped sense RNA was synthesized using the mMESSAGE mMACHINE kit (Ambion) from *pCS2+−zip7*. Microinjections were carried out using the Harvard Apparatus PLI-90 microinjector. For double *zip7*-MO injections and *pCS2+−zip7* mRNA and *zip7*-MO injections, embryos were injected separately with 1 nL of each at appropriate concentration.

### GFP reporter assay

The *zip7*-MO designed against *zip7* targeted the *zip7* ATG start site to block its translation. To assay the effectiveness of *zip7*-MO, a 195-bp zip7 cDNA fragment (27 bp upstream and 165 bp downstream of ATG start site) was fused with GFP cDNA and cloned into PCS2+ vector. The zip7-GFP reporter construct, which contains the *zip7*-MO target site, was injected to one-cell stage, wild type embryos together with or without zip7-MO. GFP was detected by fluorescent microscope at 24 hours after injection.

### Whole mount *in situ* hybridization

Antisense RNA probes were synthesized with DIG RNA Labeling Kit (AP6/T7) (Roche) from the cDNA in the pCS2+ vector. Embryos were selected at 12 hpf, 24 hpf, 36 hpf, 48 hpf, 60 hpf, or 72 hpf. Embryos beyond 24 hpf were treated with 0.003% phenylthiourea to prevent melanization, and all the embryos were removed from chorions. The steps of whole mount *in situ* hybridization referred to Sun Y [56]. Finally, the embryos were photographed using the Nikon SMZ1500 Zoom Stereomicroscope.

### Full scale scanning of zebrafish by SR-XPF

We detected the distribution of zinc in whole embryos injected with *zip7*-MO or not, and cultured with zinc or not, by SR-XRF. The absolute contents and distribution of zinc in zebrafish embryos 72 hpf were analyzed with SR-μXPF at the beamilin BL15U at Shanghai Synchrotron Radiation Facility (Shanghai, China). The continuous synchrotron X-rays were monochromatized by a Si double crystal [44]. A monochromatic X-ray beam with photon energy of 12 keV was used to excite the zebrafish. The zinc distribution in the zebrafish was continuously scanned at a step of 30 µm for both x and y directions. Each spot was irradiated for 1.5 s. The results were analyzed using the Igor Pro Folder program and Originlab OriginPro 8.5 software.

## References

[1] Andreini C, Bertini I (2011) A bioinformatics view of zinc enzymes. J Inorg Biochem. 10.1016/j.jinorgbio.2011.11.020 22209023

[2] Andreini C, Banci L, Bertini I, Rosato A (2006) Counting the zinc-proteins encoded in the human genome. J Proteome Res 5: 196–201. 16396512 10.1021/pr050361j

[3] Fukada T, Kambe T (2011) Molecular and genetic features of zinc transporters in physiology and pathogenesis. Metallomics 3: 662–674. 21566827 10.1039/c1mt00011j

[4] Prasad AS (1995) Zinc: an overview. Nutrition 11: 93–99. 7749260

[5] Fukada T, Yamasaki S, Nishida K, Murakami M, Hirano T (2011) Zinc homeostasis and signaling in health and diseases: Zinc signaling. J Biol Inorg Chem 16: 1123–1134. 21660546 10.1007/s00775-011-0797-4 PMC3176402

[6] Vallee BL, Falchuk KH (1993) The biochemical basis of zinc physiology. Physiol Rev 73: 79–118. 8419966 10.1152/physrev.1993.73.1.79

[7] Prasad AS (2009) Zinc: role in immunity, oxidative stress and chronic inflammation. Curr Opin Clin Nutr Metab Care 12: 646–652. 19710611 10.1097/MCO.0b013e3283312956

[8] Haase H, Rink L (2009) Functional significance of zinc-related signaling pathways in immune cells. Annu Rev Nutr 29: 133–152. 19400701 10.1146/annurev-nutr-080508-141119

[9] Doerr TD, Marks SC, Shamsa FH, Mathog RH, Prasad AS (1998) Effects of zinc and nutritional status on clinical outcomes in head and neck cancer. Nutrition 14: 489–495. 9646288 10.1016/s0899-9007(98)00036-7

[10] Prasad AS (2001) Recognition of zinc-deficiency syndrome. Nutrition 17: 67–69. 11165897 10.1016/s0899-9007(00)00469-x

[11] Sehgal VN, Jain S (2000) Acrodermatitis enteropathica. Clin Dermatol 18: 745–748. 11173209 10.1016/s0738-081x(00)00150-4

[12] Eide DJ (2004) The SLC39 family of metal ion transporters. Pflugers Arch 447: 796–800. 12748861 10.1007/s00424-003-1074-3

[13] Palmiter RD, Huang L (2004) Efflux and compartmentalization of zinc by members of the SLC30 family of solute carriers. Pflugers Arch. 2003/05/16 ed. pp. 744–751. 10.1007/s00424-003-1070-7 12748859

[14] Eide DJ (2006) Zinc transporters and the cellular trafficking of zinc. Biochim Biophys Acta 1763: 711–722. 16675045 10.1016/j.bbamcr.2006.03.005

[15] Liuzzi JP, Bobo JA, Cui L, McMahon RJ, Cousins RJ (2003) Zinc transporters 1, 2 and 4 are differentially expressed and localized in rats during pregnancy and lactation. J Nutr 133: 342–351. 12566465 10.1093/jn/133.2.342

[16] Lichten LA, Cousins RJ (2009) Mammalian zinc transporters: nutritional and physiologic regulation. Annu Rev Nutr 29: 153–176. 19400752 10.1146/annurev-nutr-033009-083312

[17] Taylor KM, Nicholson RI (2003) The LZT proteins; the LIV-1 subfamily of zinc transporters. Biochim Biophys Acta 1611: 16–30. 12659941 10.1016/s0005-2736(03)00048-8

[18] Huang L, Gitschier J (1997) A novel gene involved in zinc transport is deficient in the lethal milk mouse. Nat Genet 17: 292–297. 9354792 10.1038/ng1197-292

[19] Chowanadisai W, Lonnerdal B, Kelleher SL (2006) Identification of a mutation in SLC30A2 (ZnT-2) in women with low milk zinc concentration that results in transient neonatal zinc deficiency. J Biol Chem 281: 39699–39707. 17065149 10.1074/jbc.M605821200

[20] Inoue K, Matsuda K, Itoh M, Kawaguchi H, Tomoike H, et al (2002) Osteopenia and male-specific sudden cardiac death in mice lacking a zinc transporter gene, Znt5. Hum Mol Genet 11: 1775–1784. 12095919 10.1093/hmg/11.15.1775

[21] Suzuki T, Ishihara K, Migaki H, Nagao M, Yamaguchi-Iwai Y, et al (2005) Two different zinc transport complexes of cation diffusion facilitator proteins localized in the secretory pathway operate to activate alkaline phosphatases in vertebrate cells. J Biol Chem 280: 30956–30962. 15994300 10.1074/jbc.M506902200

[22] Ohana E, Hoch E, Keasar C, Kambe T, Yifrach O, et al (2009) Identification of the Zn2+ binding site and mode of operation of a mammalian Zn2+ transporter. J Biol Chem 284: 17677–17686. 19366695 10.1074/jbc.M109.007203 PMC2719407

[23] Kawasaki E (2012) ZnT8 and type 1 diabetes [Review]. Endocr J. 10.1507/endocrj.ej12-0069 22447136

[24] Rungby J (2010) Zinc, zinc transporters and diabetes. Diabetologia 53: 1549–1551. 20490449 10.1007/s00125-010-1793-x

[25] Hogstrand C, Kille P, Nicholson RI, Taylor KM (2009) Zinc transporters and cancer: a potential role for ZIP7 as a hub for tyrosine kinase activation. Trends Mol Med 15: 101–111. 19246244 10.1016/j.molmed.2009.01.004

[26] Wang F, Kim BE, Dufner-Beattie J, Petris MJ, Andrews G, et al (2004) Acrodermatitis enteropathica mutations affect transport activity, localization and zinc-responsive trafficking of the mouse ZIP4 zinc transporter. Hum Mol Genet 13: 563–571. 14709598 10.1093/hmg/ddh049

[27] Dufner-Beattie J, Wang F, Kuo YM, Gitschier J, Eide D, et al (2003) The acrodermatitis enteropathica gene ZIP4 encodes a tissue-specific, zinc-regulated zinc transporter in mice. J Biol Chem 278: 33474–33481. 12801924 10.1074/jbc.M305000200

[28] Kim BE, Wang F, Dufner-Beattie J, Andrews GK, Eide DJ, et al (2004) Zn2+-stimulated endocytosis of the mZIP4 zinc transporter regulates its location at the plasma membrane. J Biol Chem 279: 4523–4530. 14612438 10.1074/jbc.M310799200

[29] Wang F, Kim BE, Petris MJ, Eide DJ (2004) The mammalian Zip5 protein is a zinc transporter that localizes to the basolateral surface of polarized cells. J Biol Chem 279: 51433–51441. 15322118 10.1074/jbc.M408361200

[30] Weaver BP, Dufner-Beattie J, Kambe T, Andrews GK (2007) Novel zinc-responsive post-transcriptional mechanisms reciprocally regulate expression of the mouse Slc39a4 and Slc39a5 zinc transporters (Zip4 and Zip5). Biol Chem 388: 1301–1312. 18020946 10.1515/BC.2007.149 PMC2376820

[31] Yamashita S, Miyagi C, Fukada T, Kagara N, Che YS, et al (2004) Zinc transporter LIVI controls epithelial-mesenchymal transition in zebrafish gastrula organizer. Nature 429: 298–302. 15129296 10.1038/nature02545

[32] Yamashita S, Miyagi C, Carmany-Rampey A, Shimizu T, Fujii R, et al (2002) Stat3 Controls Cell Movements during Zebrafish Gastrulation. Dev Cell 2: 363–375. 11879641 10.1016/s1534-5807(02)00126-0

[33] Heldin CH, Miyazono K, ten Dijke P (1997) TGF-beta signalling from cell membrane to nucleus through SMAD proteins. Nature 390: 465–471. 9393997 10.1038/37284

[34] Taylor KM, Morgan HE, Johnson A, Nicholson RI (2004) Structure-function analysis of HKE4, a member of the new LIV-1 subfamily of zinc transporters. Biochem J 377: 131–139. 14525538 10.1042/BJ20031183 PMC1223853

[35] Colvin RA, Bush AI, Volitakis I, Fontaine CP, Thomas D, et al (2008) Insights into Zn2+ homeostasis in neurons from experimental and modeling studies. Am J Physiol Cell Physiol 294: C726–742. 18184873 10.1152/ajpcell.00541.2007

[36] Huang L, Kirschke CP, Zhang Y, Yu YY (2005) The ZIP7 gene (Slc39a7) encodes a zinc transporter involved in zinc homeostasis of the Golgi apparatus. J Biol Chem 280: 15456–15463. 15705588 10.1074/jbc.M412188200

[37] Taylor KM, Hiscox S, Nicholson RI, Hogstrand C, Kille P (2012) Protein kinase CK2 triggers cytosolic zinc signaling pathways by phosphorylation of zinc channel ZIP7. Sci Signal 5: ra11. 22317921 10.1126/scisignal.2002585 PMC3428905

[38] Stathakis DG, Burton DY, McIvor WE, Krishnakumar S, Wright TR, et al (1999) The catecholamines up (Catsup) protein of Drosophila melanogaster functions as a negative regulator of tyrosine hydroxylase activity. Genetics 153: 361–382. 10471719 10.1093/genetics/153.1.361 PMC1460756

[39] Lasswell J, Rogg LE, Nelson DC, Rongey C, Bartel B (2000) Cloning and characterization of IAR1, a gene required for auxin conjugate sensitivity in Arabidopsis. Plant Cell 12: 2395–2408. 11148286 10.1105/tpc.12.12.2395 PMC102226

[40] Feeney GP, Zheng D, Kille P, Hogstrand C (2005) The phylogeny of teleost ZIP and ZnT zinc transporters and their tissue specific expression and response to zinc in zebrafish. Biochim Biophys Acta 1732: 88–95. 16500426 10.1016/j.bbaexp.2005.12.002

[41] Lai F, Stubbs L, Lehrach H, Huang Y, Yeom Y, et al (1994) Genomic organization and expressed sequences of the mouse extended H-2K region. Genomics 23: 338–343. 7835882 10.1006/geno.1994.1509

[42] Ando A, Kikuti YY, Shigenari A, Kawata H, Okamoto N, et al (1996) cDNA cloning of the human homologues of the mouse Ke4 and Ke6 genes at the centromeric end of the human MHC region. Genomics 35: 600–602. 8812499 10.1006/geno.1996.0405

[43] Bill BR, Petzold AM, Clark KJ, Schimmenti LA, Ekker SC (2009) A primer for morpholino use in zebrafish. Zebrafish 6: 69–77. 19374550 10.1089/zeb.2008.0555 PMC2776066

[44] Wang HA, Grolimund D, Van Loon LR, Barmettler K, Borca CN, et al (2011) Quantitative chemical imaging of element diffusion into heterogeneous media using laser ablation inductively coupled plasma mass spectrometry, synchrotron micro-X-ray fluorescence, and extended X-ray absorption fine structure spectroscopy. Anal Chem 83: 6259–6266. 21623637 10.1021/ac200899x

[45] Punshon T, Guerinot ML, Lanzirotti A (2009) Using synchrotron X-ray fluorescence microprobes in the study of metal homeostasis in plants. Ann Bot 103: 665–672. 19182222 10.1093/aob/mcn264 PMC2707871

[46] Wang D, Jao LE, Zheng N, Dolan K, Ivey J, et al (2007) Efficient genome-wide mutagenesis of zebrafish genes by retroviral insertions. Proc Natl Acad Sci U S A 104: 12428–12433. 17640903 10.1073/pnas.0705502104 PMC1924792

[47] Urbanczyk A, Junemann A, Enz R (2011) PKCzeta-interacting protein ZIP3 is generated by intronic polyadenylation, and is expressed in the brain and retina of the rat. Biochem J 433: 43–50. 20979579 10.1042/BJ20101111

[48] Chowanadisai W, Lonnerdal B, Kelleher SL (2008) Zip6 (LIV-1) regulates zinc uptake in neuroblastoma cells under resting but not depolarizing conditions. Brain Res 1199: 10–19. 18272141 10.1016/j.brainres.2008.01.015

[49] Qian L, Lopez V, Seo YA, Kelleher SL (2009) Prolactin regulates ZNT2 expression through the JAK2/STAT5 signaling pathway in mammary cells. Am J Physiol Cell Physiol 297: C369–377. 19494234 10.1152/ajpcell.00589.2008 PMC2724096

[50] Guo L, Lichten LA, Ryu MS, Liuzzi JP, Wang F, et al (2010) STAT5-glucocorticoid receptor interaction and MTF-1 regulate the expression of ZnT2 (Slc30a2) in pancreatic acinar cells. Proc Natl Acad Sci U S A 107: 2818–2823. 20133611 10.1073/pnas.0914941107 PMC2840329

[51] Fukunaka A, Suzuki T, Kurokawa Y, Yamazaki T, Fujiwara N, et al (2009) Demonstration and characterization of the heterodimerization of ZnT5 and ZnT6 in the early secretory pathway. J Biol Chem 284: 30798–30806. 19759014 10.1074/jbc.M109.026435 PMC2781478

[52] Hirano T, Murakami M, Fukada T, Nishida K, Yamasaki S, et al (2008) Roles of zinc and zinc signaling in immunity: zinc as an intracellular signaling molecule. Adv Immunol 97: 149–176. 18501770 10.1016/S0065-2776(08)00003-5

[53] Taylor KM, Vichova P, Jordan N, Hiscox S, Hendley R, et al (2008) ZIP7-mediated intracellular zinc transport contributes to aberrant growth factor signaling in antihormone-resistant breast cancer Cells. Endocrinology 149: 4912–4920. 18583420 10.1210/en.2008-0351

[54] Kimmel CB, Ballard WW, Kimmel SR, Ullmann B, Schilling TF (1995) Stages of embryonic development of the zebrafish. Dev Dyn 203: 253–310. 8589427 10.1002/aja.1002030302

[55] Ho E, Dukovcic S, Hobson B, Wong CP, Miller G, et al (2012) Zinc transporter expression in zebrafish (Danio rerio) during development. Comp Biochem Physiol C Toxicol Pharmacol 155: 26–32. 21596156 10.1016/j.cbpc.2011.05.002 PMC3196795

[56] Sun Y, Sun BC, Liu YX, Zhang SW, Zhao XL, et al (2008) [Diagnostic value of SYT-SSX fusion gene detection by fluorescence in-situ hybridization for synovial sarcoma]. Zhonghua Bing Li Xue Za Zhi 37: 660–664. 19094483

